# Epoxylathyrane Derivatives as MDR-Selective Compounds for Disabling Multidrug Resistance in Cancer

**DOI:** 10.3389/fphar.2020.00599

**Published:** 2020-05-08

**Authors:** Mariana Alves Reis, Ana M. Matos, Noélia Duarte, Omar Bauomy Ahmed, Ricardo J. Ferreira, Hermann Lage, Maria-José U. Ferreira

**Affiliations:** ^1^Faculty of Pharmacy, Research Institute for Medicines (iMed.ULisboa), Universidade de Lisboa, Lisbon, Portugal; ^2^Institute of Pathology, University Hospital Charité, Berlin, Germany; ^3^Science for Life Laboratory, Department of Cell and Molecular Biology, Uppsala University, Uppsala, Sweden

**Keywords:** multidrug resistance, collateral sensitivity, apoptosis, *Euphorbia*, macrocyclic diterpenes, lathyrane, regression models

## Abstract

**Background:**

Multidrug resistance (MDR) has been regarded as one of the major hurdles for the successful outcome of cancer chemotherapy. The collateral sensitivity (CS) effect is one the most auspicious anti-MDR strategies. Epoxylathyrane derivatives **1–16** were obtained by derivatization of the macrocyclic diterpene epoxyboetirane A (**17**), a lathyrane-type macrocyclic diterpene isolated from *Euphorbia boetica*. Some of these compounds were found to strongly modulate P-glycoprotein (P-gp/ABCB1) efflux.

**Purpose:**

The main goal was to develop lathyrane-type macrocyclic diterpenes with improved MDR-modifying activity, by targeting more than one anti-MDR mechanism.

**Study design/methods:**

In this study, the potential CS effect of compounds **1**–**16** was evaluated against gastric (EPG85-257), pancreatic (EPP85-181), and colon (HT-29) human cancer cells and their drug-resistant counterparts, respectively selected against mitoxantrone (EPG85-257RNOV; EPP85-181RNOV; HT-RNOV) or daunorubicin (EPG85-257RDB; EPP85-181RDB; HT-RDB). The most promising compounds (**8**, **15**, and **16**) were investigated as apoptosis inducers, using the assays annexin V/PI and active caspase-3.

**Results:**

The compounds were more effective against the resistant gastric cell lines, being the CS effect more significant in EPG85-257RDB cells. Taking together the IC_50_ values and the CS effect, compounds **8**, **15**, and **16** exhibited the best results. Epoxyboetirane P (**8**), with the strongest MDR-selective antiproliferative activity against gastric carcinoma EPG85-257RDB cells (IC_50_ of 0.72 µM), being 10-fold more active against this resistant subline than in sensitive gastric carcinoma cells. The CS effect elicited by compounds **15** and **16** appeared to be by inducing apoptosis *via* caspase-3 activation. Structure-activity relationships of the compounds were additionally obtained through regression models to clarify the structural determinants associated to the CS effect.

**Conclusions:**

This study reinforces the importance of lathyrane-type diterpenes as lead molecules for the research of MDR-modifying agents.

## Introduction

Multidrug resistance (MDR) is among the main clinical hurdles to successful cancer chemotherapy. It is defined by the development of cell resistance to a large variety of structurally unrelated drugs with diverse mechanisms of action. There is a great consensus that cancer cells might become resistant to anticancer drugs by several mechanisms that are still not completely understood and could occur simultaneously. Some of the most common cellular factors attributed to MDR include: changes in membrane transport through reduced drug uptake or augmented drug efflux; changes in drug targets and metabolism; increased DNA damage repair; and failure of apoptotic events. However, the most known and characterized MDR mechanism is owing to an increased efflux of the anticancer drugs as a result of the overexpression of ATP-binding cassette (ABC) transporter proteins, namely, P-glycoprotein (P-gp/ABCB1), multidrug resistance protein 1 (MRP1/ABCC1), and breast cancer resistance protein (BCRP/ABCG2) that act as extrusion pumps. From these mechanisms, P-gp still constitutes one of the biggest challenges for medicinal chemistry (Gottesman et al., 2002; Gottesman and Ling, 2006; Gottesman et al., 2016).

Several approaches have been developed to eradicate MDR in cancer. The most general has been the development of P-gp inhibitors to co-administer with anticancer drugs. However, despite great *in vitro* success, there is no P-gp inhibitor currently available for clinical use. The development of collateral sensitizing compounds is also included on the set of the most encouraging approaches to tackle MDR (Callaghan et al., 2014; Szakács et al., 2014). The collateral sensitivity (CS) effect is characterized by an increased sensitivity or hypersensitivity of resistant cells to certain compounds. The phenomenon was firstly recognized in the early 1950s, when it was observed that resistant *Escherichia coli* was hypersensitive to several drugs at the same time (Szybalski and Bryson, 1952). Based on this concept, which presently is considered a strong anti-MDR strategy, alterations of cancer cells that confer resistance to certain agents might simultaneously generate weaknesses that may rend drug-resistant cells more sensitive to alternative drugs than the corresponding parental cells. CS is thought to be highly correlated with the overexpression of one of the three major efflux proteins (P-gp, MRP1 or BCRP) in resistant cancer cells, therefore representing a new strategy to circumvent ABC transporters-mediated MDR (Gottesman et al., 2016). Thus, these vulnerabilities developed by cancer cells can be targeted for improving chemotherapy by developing compounds that are selective against resistant-phenotypes (MDR-selective compounds) and thus able to re-sensitize resistant tumors and reestablish drugs effectiveness. Several compounds have been reported to exert CS. However, although many hypotheses have been proposed, the mechanism of collateral sensitizing compounds remains unclear (Callaghan et al., 2014; Szakács et al., 2014). In this way, while in P-gp-overexpressing cancer cells, CS agents seem to be related to diverse biochemical mechanisms, in MRP1-overexpressing cancer cells, CS agents appeared to behave as stimulators of glutathione efflux, altering redox balance and thus triggering apoptosis of multi-resistant cells (Klukovits and Krajcsi, 2015).

Natural products have been of crucial importance for drug research and development. Concerning cancer, since the beginning of chemotherapy in the 1940s to date, about 75% of anticancer drugs approved world-wide are natural products or their synthetic derivatives (Newman and Cragg, 2012). In an effort to find out anticancer compounds from plants, for targeting MDR cancer cells, our group has been given particular attention on the development of MDR reversal compounds (Reis et al., 2015; Paterna et al., 2016; Ramalhete et al., 2016; Reis et al., 2016; Reis et al., 2017; Ferreira et al., 2018a; Ferreira et al., 2018b; Paterna et al., 2018; Ramalhete et al., 2018). Duo to the high and unusual chemical diversity of their metabolites, many of which coupled with strong biological properties, we have given particular attention to *Euphorbia* species (Euphorbiaceae), which are well known since ancient times for their use in folk medicine worldwide, namely, to cure cancer, tumors, and warts (Hartwell, 1969; Ernst et al., 2015). Other significant reported uses included treatment of respiratory and digestive disorders and inflammation (Ernst et al., 2015). Most importantly, in 2012, Food and Drug Administration (FDA) and European Medicines Agency (EMA) approved ingenol 3-angelate (Picato^®^), isolated from *Euphorbia peplus*, for the treatment of actinic keratosis. This diterpene ester, with a dual and unique mechanism of action embracing a rapid cellular necrosis and a specific immune response (Rosen et al., 2012), is a valuable example of the strong bioactivity and pharmacological importance of some *Euphorbia* genus metabolites.

Jatrophane and lathyrane-type diterpenes, from *Euphorbia* species, have revealed a significant MDR modulatory activity through reversion of the ABCB1 MDR phenotype (Ferreira et al., 2014). Aiming at optimizing their structures for improving their MDR reversal activity, *in silico* and structure-activity relationship studies were performed (Ferreira et al., 2011; Reis et al., 2012; Sousa et al., 2012; Ferreira et al., 2013; Reis et al., 2013; Baptista et al., 2016). In this regard, *Euphorbia boetica* Boiss. (Euphorbiacae) was a fruitful source of novel compounds and prototypes for the design of MDR reversers (Vieira et al., 2014; Matos et al., 2015). Macrocyclic diterpene derivatives with the lathyrane skeleton, obtained from this species, were found promising ABCB1 efflux modulators (Vieira et al., 2014; Matos et al., 2015).

**Figure 1:**
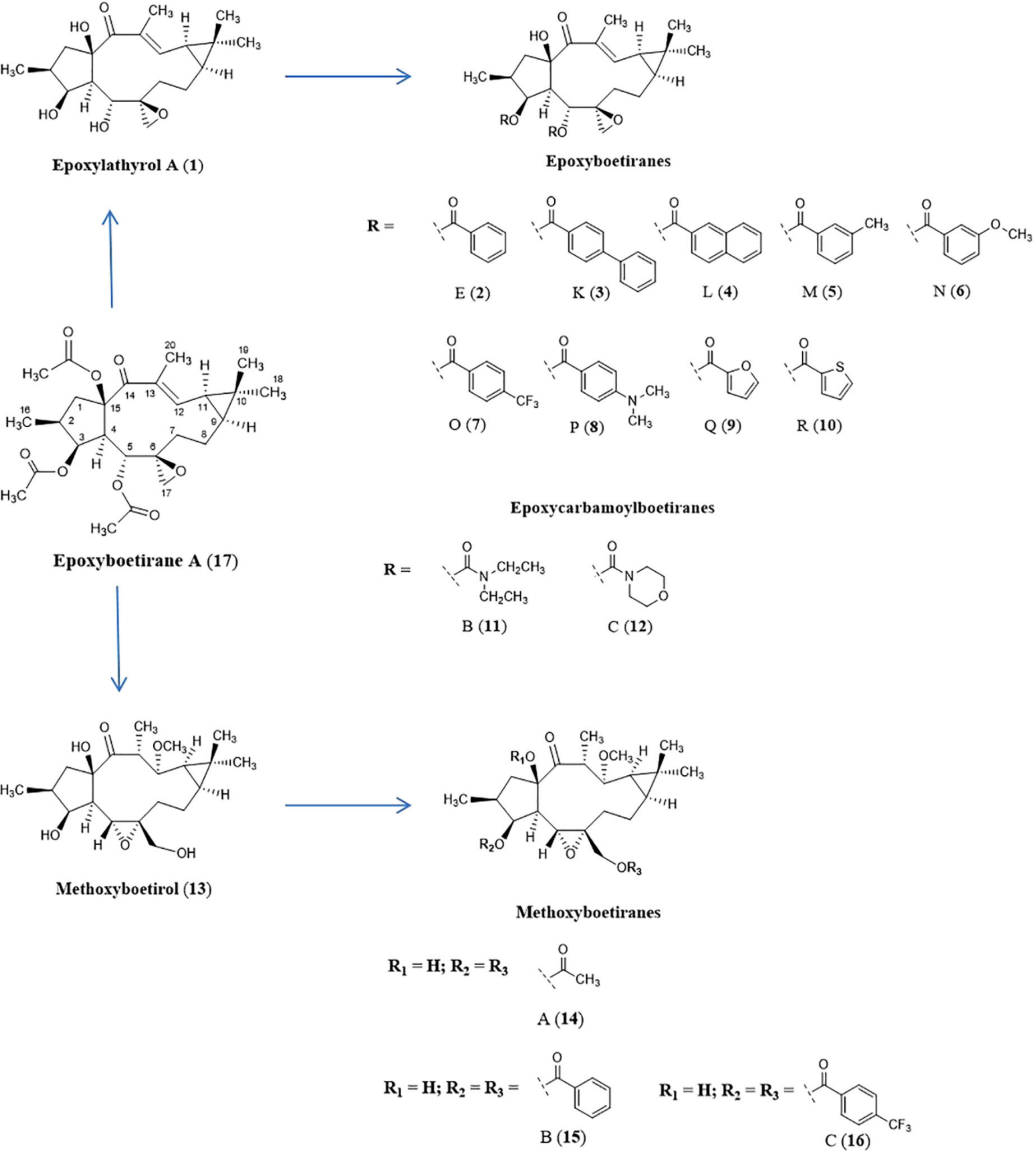
Structures of compounds **1**–**17**.

Therefore, the present work aimed at assessing the ability of the macrocyclic diterpene derivatives **1–16** (**Figure 1**) for their potential as collateral sensitizing compounds, using the human tumor gastric (EPG85-257), pancreatic (EPP85-181), and colon (HT-29) cell models (drug-sensitive and drug-resistant sublines), well described for MDR (**Table S2**; Lage et al., 2010; Hilgeroth et al., 2013; Reis et al., 2014). Additionally, the MDR-selective antiproliferative activity mode of action of compounds **8**, **15**, and **16** was assessed towards apoptosis and caspase-3 activation, using the same cell lines. From the obtained results, and to better understand which structural features are correlated with the observed CS effect, regression models were further obtained from molecular descriptors calculated for a small library of macrocyclic diterpenes.

## Materials and Methods

### Tested Compounds

Epoxylathyrane derivatives (**1–16**), namely, epoxylathyrol (**1**), epoxyboetirane E (**2**), epoxyboetirane L (**4**), epoxyboetirane M (**5**), epoxyboetirane N (**6**), epoxyboetirane O (**7**), epoxyboetirane P (**8**), epoxyboetirane Q (**9**), epoxyboetirane R (**10**), epoxycarbamoylboetirane B (**11**), epoxycarbamoylboetirane C (**12**), methoxyboetirane B (**15**), and methoxyboetirane C (**16**), were obtained by derivatization of the macrocyclic diterpene epoxyboetirane A (**17**), isolated from *Euphorbia boetica* (Vieira et al., 2014; Matos et al., 2015). Briefly, epoxyboetirane A, was firstly hydrolyzed, yielding mostly epoxylathyrol (**1**). Methoxyboetirol (**13**), resulting from a Payne-rearranged Michael adduct, was also obtained as minor product. Compound **2–12** and **14**–**16** were obtained by acylation reactions of epoxylathyrol (**1**) and methoxyboetirol (**13**), respectively (Vieira et al., 2014; Matos et al., 2015).

### Cell Lines, Cell Culture

The establishment and characterization of the cell lines EPG85-257P (gastric); EPP85-181P (pancreatic); and HT-29P (colon) and their drug-resistant sublines (EPG85-257RNOV, EPG85-257RDB, EPP85-181RNOV, EPP85-181RDB, HT-29RNOV, HT-29RDB used have been described previously (Reis et al., 2014). More experimental details at supplementary material and **Table S2**.

### Cell Proliferation Assay, Annexin V/PI Staining, and Active Caspase-3 Assay

The antiproliferative activity of compounds was evaluated using a proliferation assay based on sulforhodamine B (SRB) staining, as previously described (Reis et al., 2014). More experimental details at supplementary material.

For detection of cytotoxic drug-induced apoptosis, a FITC Annexin V apoptosis detection kit (BD Pharmingen, BD Biosciences) was used. Detection of intracellular presence of active caspase-3 was also performed using FITC active Caspase-3 Apoptosis Kit (BD Pharmingen, BD Biosciences). Both assays followed the same experimental design, described at supplementary material.

### Regression Models

For a given set of molecules obtained in the present and previous works (*N* = 42, **Table S1**) (Reis et al., 2014; Reis et al., 2016; Reis et al., 2017), a comprehensive database of molecular descriptors (constitutional, topological, and geometrical) was obtained from E-DRAGON, PaDEL, and MOE as in a previous work (Baptista et al., 2016). Afterward, relative resistant (RR) values obtained in the EPG-257RDB cell line were added to the dataset and transformed into a binary classification of 1 (RR ≤ 0.5) or 0 (RR > 0.5) for the presence or absence of CS, respectively. All QSAR models were built using WEKA v3.8.3 (Hall et al., 2009) software as previously reported (Baptista et al., 2016), with only minor modifications. The CfsSubsetEval algorithm with the BestFirst search method was applied as default prior to model classification. All models were generated using WEKA’s default options. The robustness of the generated models (NB and RT) were assessed by a 10-fold cross-validation and their predictive power by splitting the dataset into training and test sets (66:34). All models were analysed using several parameters, among which true positive rate (TP), false positive rate (FP), precision, Matthews Correlation Coefficient (MCC), and Receiver Operating Characteristics (ROC) area under the curve. Other parameters as the mean absolute error (MAE), root mean squared error (RMSE), and Kappa statistic (*k*) were also used to assess the reliability of the model.

## Results and Discussion

### Collateral Sensitivity Effect

Aiming at optimizing macrocyclic diterpenes with lathyrane scaffold as active MDR reversal agents, the phytochemical investigation of *Euphorbia boetica* aerial parts (methanolic extract) was conducted, yielding several compounds with the lathyrane scaffold (Vieira et al., 2014). Epoxylathyrane A (**17**), a lathyrane polyester isolated in large amount, was hydrolyzed, in an alkaline methanolic solution, giving rise to epoxylathyrol (**1**), as main product, and a Payne-rearranged Michael adduct named methoxyboetirol (**13**) (Matos et al., 2015). In this previous work, compounds **1** and **13** were acylated, yielding the derivatives **2**–**12** and **14**–**16**, respectively. The potential of epoxylathyrane derivatives **1**–**16** as P-gp-mediated MDR reversers, was evaluated at non-cytotoxic doses in L5178Y *ABCB1*-transfected mouse T-lymphoma cells, by the accumulation rhodamine-123 assay. Most of the tested derivatives exhibited strong P-gp modulating activities and, in addition, they were able to enhance the cytotoxicity of doxorubicin in a synergistic mode, restoring its sensitivity by reversion of the ABCB1-MDR phenotype. By structure-activity relationships studies, it was concluded that the presence of an aromatic moiety on those structures improved significantly the inhibition of rhodamine-123 efflux (Matos et al., 2015).

Taking into account that targeting more than one MDR mechanism could be a way to provide a good therapeutic outcome, in the course of the development of epoxylathyrane derivatives as MDR reversal agents, it is also important to consider other potential anti-MDR mechanisms of action (Fojo, 2008; Gottesman et al., 2016). Therefore, the epoxylathyrane derivatives **1–16** were investigated for their potential CS effect against gastric (EPG85-257), pancreatic (EPP85-181), and colon (HT-29) human cancer cells and their drug-resistant counterparts, respectively selected against mitoxantrone (RNOV) or daunorubicin (RDB), using a proliferation assay (Reis et al., 2014). The MDR-selective activity was assessed by the relative resistance ratio (RR = IC_50(resistant)_/IC_50(parental)_). Values of RR < 1 indicate that the compound kills MDR cells more effectively than parental cells, but if RR ≤ 0.5, then a CS effect is taking place (Hall et al., 2009). The cytotoxic agents etoposide and cisplatin were used as positive controls. The antiproliferative activity and CS effects of compounds **1–16** are presented in **Tables 1**–**4**. The heat map represented in **Table 1** allows the recognition of compounds that exhibit MDR-selective activity at a specific IC_50_ level.

**Table 1:** Heat map table summarizing the antiproliferative and collateral sensitivity results against gastric carcinoma cells (EPG85-257P, EPG85-257RNOV, and EPG85-257RDB), and pancreatic carcinoma cells (EPP85-181P, EPP85-181RNOV, and EPP85-181RDB). This representation allows finding compounds that present MDR-selective activity at a determined IC_50_ level. CS values (RR ≤ 0.5) are presented.

	257P	257RNOV	257RDB	181P	181RNOV	181RDB	HT-29P	HT-29NOV	HT-29RDB			
Epoxylathyrol (**1**)												
Epoxyboetirane E (**2**)			0.32									IC_50:_
Epoxyboetirane K (**3**)												> 30 μM
Epoxyboetirane L (**4**)		0.38	0.21									10-30 μM
Epoxyboetirane M (**5**)		0.42	0.19									< 10 μM
Epoxyboetirane N (**6**)		0.27	0.12									
Epoxyboetirane O (**7**)		0.21										
Epoxyboetirane P (**8**)			0.09									
Epoxyboetirane Q (**9**)			0.28									
Epoxyboetirane R (**10**)			0.31									
Epoxycarbamoylboetirane B (**11**)			0.03									
Epoxycarbamoylboetirane C (**12**)												
Methoxyboetirol (**13**)												
Methoxyboetirane A (**14**)												
Methoxyboetirane B (**15**)		0.47	0.27									
Methoxyboetirane C (**16**)			0.39									

**Table 2:** Antiproliferative activity of compounds **1–16** in gastric carcinoma cells: EPG85-257P (parental), EPG85-257RNOV (multidrug resistance [MDR] phenotype), and EPG85-257RDB (multidrug resistance [MDR] phenotype).

Compound	EPG85-257P	EPG85-257RNOV		EPG85-257RDB	
	IC_50_ ± SD (μM)	IC_50_ ± SD (μM)	RR^a^	IC_50_ ± SD (μM)	RR^a^
Epoxylathyrol (**1**)	>100	> 100	—	70.53 ± 6.24	< 0.70
Epoxyboetirane E (**2**)	19.67 ± 1.56	9.97 ± 0.38	0.50	6.40 ± 0.68	0.32
Epoxyboetirane L (**4**)	>100	38.17 ± 2.39	<0.38	21.13±1.76	< 0.21
Epoxyboetirane M (**5**)	>25 ^b^	10.53 ± 0.44	<0.42	4.74 ± 0.21	< 0.19
Epoxyboetirane N (**6**)	39.67 ± 4.88	10.62 ± 0.64	0.27	4.56 ± 0.47	0.12
Epoxyboetirane O (**7**)	>100	21.41 ± 1.35	<0.21	50.91 ± 4.95	< 0.50
Epoxyboetirane P (**8**)	7.81 ± 2.01	5.91 ± 1.15	0.76	0.72 ± 0.08	0.09
Epoxyboetirane Q (**9**)	>25 ^b^	>25 ^b^	—	7.15 ± .1.24	< 0.28
Epoxyboetirane R (**10**)	21.08. ± 2.53	10.73 ± 1.36	0.50	6.63 ± 0.97	0.31
Epoxycarbamoylboetirane B (**11**)	>100	78.13 ± 3.69	<0.78	2.59 ± 0.39	< 0.03
Methoxyboetirane B (**15**)	12.52 ± 1.15	5.89 ± 0.62	0.47	3.39 ± 0.44	0.27
Methoxyboetirane C (**16**)	10.12 ± 0.68	8.25 ± 1.49	0.82	3.98 ± 0.31	0.39
Etoposide	0.105 ± 0.0	1.55 ± 0.1	14.8	6.2 ± 0.3	59
Cisplatin	4.4 ± 0.4	2.6 ± 0.2	0.6	4.0 ± 0.3	1
DMSO (2%)	>100	>100	—	>100	—

^a^Relative resistance ratio, RR = IC_50(resistant)_/IC_50(parental)_); ^b^above this concentration the compound crystallized in the culture medium. For compounds **3**, **12, 13** and **14** the IC_50_ values were found to be above 100 µM against the parental and both resistant cells. Each IC_50_ value indicates the mean ± SD of n = 3 to 4 independent experiments (each concentration was performed in triplicate per experiment).

**Table 3:** Antiproliferative activity of **1–16** in in pancreatic carcinoma cells: EPP85-181P (parental), EPP85-181RNOV (multidrug resistance [MDR] phenotype), and EPP85-181RBD (multidrug resistance [MDR] phenotype).

Compound	EPP85-181P	EPP85-181RNOV		EPP85-181RDB	
	IC_50_ ± SD (μM)	IC_50_ ± SD (μM)	RR^a^	IC_50_ ± SD (μM)	RR^a^
Epoxyboetirane E (**2**)	9.52 ± 0,40	41.24 ± 3.54	4.33	13.18 ± 1.65	1.38
Epoxyboetirane M (**5**)	17.49 ± 0.08	> 25 ^b^	> 1.43	21.07 ± 2.06	1.20
Epoxyboetirane N (**6**)	20.63 ± 0.90	57.33 ± 4.90	2.78	14.17 ± 2.34	0.69
Epoxyboetirane P (**8**)	4.88 ± 0.11	5.42 ± 0.49	1.11	2.57 ± 0.30	0.53
Methoxyboetirane B (**15**)	12.08 ± 1.84	10.01 ± 0.42	0.83	10.18 ± 0.81	0.84
Methoxyboetirane C (**16**)	16.51 ± 1.97	8.72 ± 0.43	0.53	20.95 ± 1.79	1.27
Etoposide	0.58 ± 0.0	4.5 ± 0.7	7.8	62.0 ± 4.2	106.9
Cisplatin	0.08 ± 0.0	2.6 ± 0.2	34	0.09 ± 0.0	1.2
DMSO (2%)	> 100	> 100	—	> 100	—

^a^Relative resistance ratio, RR = IC_50(resistant)_/IC_50(parental)_) ^b^above this concentration the compound crystallized in the culture medium. For all the other compounds (**1 3**, **4**, **7** and **11-14**), the IC_50_ values were found to be above 100 µM, except for epoxyboetiranes Q (**9**) and R (**10**) which was above 25 µM and 50 µM, respectively, the maximum tested concentrations for each due to lower water solubility. Each IC_50_ value indicates the mean ± SD of n = 3 to 4 independent experiments (each concentration was performed in triplicate per experiment).

**Table 4:** Antiproliferative activity of compounds **1–16** in colon carcinoma cells HT-29P (parental), HT-29RNOV (multidrug resistance [MDR] phenotype), and HT-29RBD (multidrug resistance [MDR] phenotype).

Compound	HT-29P	HT-29RNOV		HT-29RDB	
	IC_50_ ± SD (μM)	IC_50_ ± SD (μM)	RR^a^	IC_50_ ± SD (μM)	RR^a^
Epoxyboetirane E (**2**)	> 50^b^	32.27 ± 4.69	< 0.65	47.21 ± 5.17	< 0.94
Epoxyboetirane N (**6**)	> 100	48.92 ± 4.92	< 0.49	31.16 ± 0.53	< 0.31
Epoxyboetirane P (**8**)	8.24 ± 0.47	5.00 ± 0.16	0.61	5.25 ± 0.07	0.64
Epoxyboetirane R (**10**)	> 50^b^	30.20 ± 4.05	< 0.60	> 50^b^	—
Methoxyboetirane B (**15**)	20.40 ± 1.83	9.40 ± 0.30	0.46	9.61 ± 0.10	0.47
Methoxyboetirane C (**16**)	17.20 ± 2.62	9.39 ± 0.37	0.55	13.12 ± 1.15	0.76
Cisplatin	3.8 ± 0.1	3.8 ± 0.1	1	2.7 ± 0.1	0.7
Etoposide	2.3 ± 0.3	35.0 ± 2.6	15.2	26.0 ± 1.7	11.3
DMSO (2%)	> 100	> 100	—	> 100	—

^a^Relative resistance ratio, RR = IC_50(resistant)_/IC_50(parental)_); ^b^Above this concentration the compound crystallized in the culture medium. For all the other compounds, the IC_50_ was found to be above 100 µM, except for epoxyboetiranes M (**5**) and Q (**9**) which was above 25 µM – the maximum tested concentration due to lower water solubility. Each IC_50_ value indicates the mean ± SD of n = 3 to 4 independent experiments (each concentration was performed in triplicate per experiment).

As showed in **Tables 2**–**4**, the strongest antiproliferative effect in drug-sensitive cell lines was observed for epoxyboetirane P (**8**), bearing *p*-(dimethylamino)benzoyl acyl groups, that exhibited an IC_50_ < 10 µM in the three cell lines tested (EPG85-257P, EPP85-181P, and HT-29P, IC_50_ = 7.81 ± 2.01, 4.88 ± 0.11, and 8.24 ± 0.47, respectively). An IC_50_ < 10 µM was also found for epoxyboetirane E (**2**) but only in pancreatic parental cells (EPP85-181P, IC_50_ = 9.52 ± 0.40). Significant IC_50_ values were also observed for methoxyboetiranes B (**15**) and C (**16**), in gastric parental cells (EPG85-257P, IC_50_ = 12.52 ± 1.15 and 10.12 ± 0.68, respectively), exhibiting the former (**15**) also an IC_50_ = 12.08 ± 1.84, against pancreatic cells. The other compounds were inactive or barely active, displaying a moderate/weak antiproliferative activity in parental drug-sensitive cell lines (**Tables 2**–**4**).

When analyzing the results against MDR sublines, several compounds were found to be more active against multidrug-resistant cells than in parental cells (**Tables 2**–**4**), exhibiting relative resistance values lower than 1.0. Moreover, as it can be observed, some compounds showed CS effect (RR ≤ 0.5), being more effective toward the resistant gastric cell lines, and more pronounced in EPG85-257RDB cells (**Tables 1** and **2**), which are characterized by ABCB1 overexpression (Dietel et al., 1990). Taking together the IC_50_ and RR values, the best results were found for epoxyboetirane P (**8**), which showed an antiproliferative effect 10-fold higher against the MDR subline of gastric carcinoma EPG85-257RDB than in parental drug-sensitive cells (IC_50_ = 0.72 ± 0.08; RR = 0.09). A selective IC_50_ value was also obtained with this compound against the resistant EPG85-257 RNOV subline (IC_50_ = 5.91 ± 1.15), although without CS effect (RR = 0.76). CS effect, associated with potent antiproliferative activity, was also registered for methoxyboetiranes B (**15**), (IC_50_ = 3.39 ± 0.44; RR = 0.27), and C (**16**) (IC_50_ = 3.98 ± 0.31; RR = 0.39), against the gastric EPG85-257 RDB subline, whose IC_50_ values were comparable to those of the positive controls etoposide and cisplatin and (IC_50_ = 6.2 ± 0.3 and 4.0 ± 0.3, respectively). Compound **15**, having unsubstituted benzoyl moieties, showed also CS effect in EPG85-257 RNOV subline (IC_50_ = 5.89 ± 0.62; RR = 0.47), whereas compound **16**, having trifluoromethyl substituents at *para*-position, exhibited slightly higher relative resistance and IC_50_ values (IC_50_ = 8.25 ± 1.49; RR = 0.82). Furthermore, epoxyboetyranes E, M, N, O, R (**2**, **5**, **6**, **9**, and **10**) and epoxycarbamoylboetirane B (**11**) also exhibited CS effect (RR ≤ 0.5) coupled with high selective antiproliferative activity (IC_50_ values ranging from 2.59 ± 0.39 to 7.15 ± 1.24) against the gastric EPG85-257 RDB subline. Excepting for compound **11**, comparable results were also observed for this set of compounds in the EPG85-257 RNOV subline, although associated with a lower antiproliferative effect (**Table 2**).

The tested compounds were less active and showed no CS effect in pancreatic cancer cell lines (**Tables 1** and **3**). Epoxyboetirane P (**8**) was once more the most active (IC_50_ < 10 µM) in parental and both resistant sublines, exhibiting MDR-selective antiproliferative effects (RR < 1) in resistant EPP85-181RRDB subline (EPP85-181P, IC_50_ = 4.88 ± 0.11; EPP85-181RDB, IC_50_ = 2.57 ± 0.30, RR = 0.53; EPP85-181RNOV, IC_50_ = 5.42 ± 0.49, RR = 1.11). When comparing with the positive controls, methoxyboetirane B (**15**) also showed significant antiproliferative activity in both resistant sublines associated with RR < 1 (EPP85-181RDB, IC_50_ = 10.18 ± 0.81, RR = 0.84; EPP85-181RNOV, IC_50_ = 10.01 ± 0.42, RR = 0.83).

In turn, the best results revealed by methoxyboetirane C (**16**) were in EPP85-181RNOV subline, showing an IC_50_ = 8.72 ± 0.43. (RR = 0.53).

Epoxyboetirane P (**8**) also showed the lowest IC_50_ values in colon cancer cell lines (**Table 4**), (HT-29RDB, IC_50_ = 5.25 ± 0.07 μM, RR = 0.64; HT-29RNOV; IC_50_ = 5.00 ± 0.16 μM, RR = 0.61). However, in colon cancer cells, CS effect (RR ≤ 5), associated with significant antiproliferative activity (IC_50_≤ 10), was only observed for methoxyboetirane B (**15**), with similar results in both resistant variants (HT-29RDB, IC_50_ = 9.61 ± 0.10, RR = 0.47; HT-29RNOV IC_50_ = 9.40 ± 0.30, RR = 0.46). Methoxyboetirane C (**16**) showed comparable IC_50_ values against HT-29RNOV variant (9.39 ± 0.37, RR = 0.55). Indeed, it exhibited RR< 1 in HT-29RDB subline although with lower antiproliferative activity (IC_50_ = 13.12 ± 1.15, RR = 0.76).

When analyzing the results, it could be concluded that the antiproliferative activity depends on the acylation patterns. Thus, in both epoxylathyrol (**1**) and methoxyboetirol (**13**) derivatives, (**2**–**12** and **13–16**, respectively), acyl moieties bearing simple aromatic moieties, including benzoyl (**2**, **5**, **6–8**, **15**, **16**) furoyl (**9**), and thiophenecarbonyl (**10**) groups, were generally favorable for the antiproliferative activity. Conversely, compounds with biphenylcarbonyl (**3**) and naphthoyl substituents (**4**) were inactive or barely active in the three human cancer parental cells and corresponding MDR-sublines. No significant activity was observed for the parent compounds epoxylathyrol (**1**) and methoxyboetirol (**13**), without ester moieties, and for compounds **11**, **12**, and **14**, bearing aliphatic acyl moieties.

As already mentioned, this set of macrocyclic diterpenes (**1**–**16**) was previously evaluated for their ability to reverse P-gp-mediated MDR, using a functional assay (Vieira et al., 2014; Matos et al., 2015). In this study, it is noteworthy that compounds with significant MDR-selective antiproliferative activities (**2**, **5**, **6**, **8**–**11**, **15**, and **16)**, mostly in drug-resistant gastric sublines, were also found to be strong P-gp modulators in a concentration-dependent manner.

### Apoptosis Induction Activity

**Figure 2:**
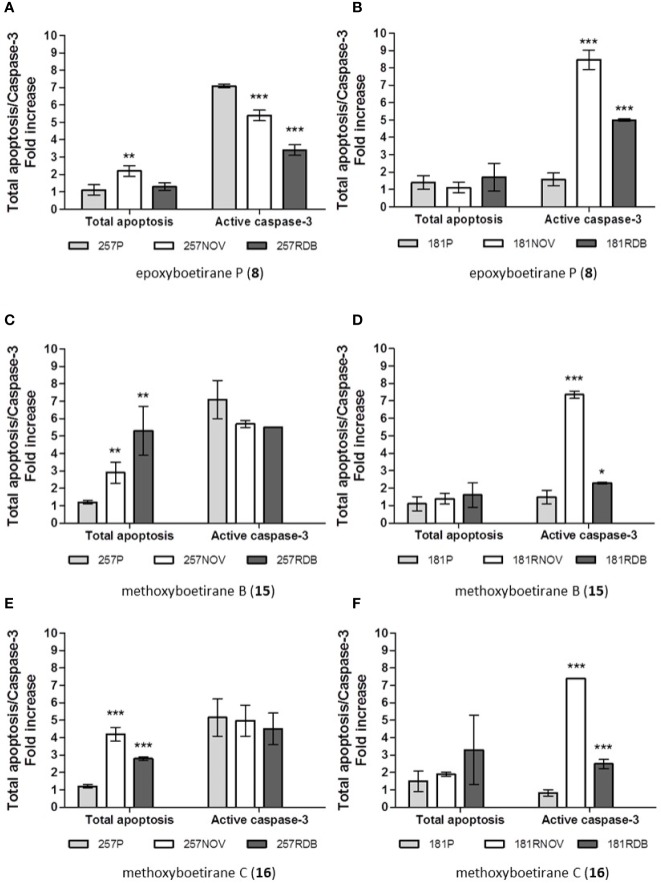
Cell death mechanism measurements: apoptosis induction and active caspase-3 activation in gastric (**A, C, E**) and pancreatic (**B, D, F**) cancer cell lines after 72 h incubation with epoxyboetirane P (**8**), methoxyboetirane B (**15**), and methoxyboetirane C (**16**) (30 μM). Apoptosis induction: representative flow cytometry analysis after annexin V-FITC/PI staining. The FL1 and FL2 axis represent the fluorescence intensities of Annexin V-FITC and PI, respectively. Camptothecin (1 μM) was used as internal positive control. Total apoptosis was considered the sum of early and late apoptotic events (cells annexin V-FITC positive/PI negative plus cells annexin V-FITC positive/PI positive). The results were expressed as the ratio between treated samples with untreated. Each column represents the mean ± SD of three independent experiments. Statistical significance was calculated for the difference between treated resistant cell lines and treated parental cells using a two-tailed unpaired Student’s t test. Level of significance *, p < 0.05. **, p < 0.01. ***, p < 0.001. Active caspase-3: the results were expressed as the ratio between treated samples with untreated. Each column represents the mean ± SD (n = 3). Statistical significance was calculated for the difference between treated resistant cell lines and treated parental cells using a two-tailed unpaired Student’s t test. Level of significance *, p < 0.05. **, p < 0.01. ***, p < 0.001.

The ability of compounds **8**, **15**, and **16** as apoptosis inducers was evaluated using as models gastric and pancreatic cancer cells. The apoptotic process usually occurs through the extrinsic or intrinsic pathways. Despite their mechanistic differences, both converge on the same execution pathway, which is initiated by the activation of caspase-3. This cysteine protease takes a fundamental part in apoptosis, being pro-caspase-3, the penultimate enzyme for accomplishment of the apoptotic process (Tan et al., 2009). Therefore, the active caspase-3 was quantified by flow cytometry after 48 h of exposure at 20 µM of the compounds **8**, **15**, **16** (**Figure 2**). The results were expressed as fold increase (ratio between treated samples and untreated samples).

The subsequent apoptotic events include exposure of phosphatidylserine on the external surface of the plasma membrane, which is used as marker of apoptosis. As this phospholipid is shifted from the inner to the outer leaflet of the plasma membrane in early apoptosis, the annexin V/propidium iodide (PI) staining permits to identify both early and late apoptotic cells (PI negative, annexin V positive, and PI positive, annexin V positive, respectively) (Chen, 2009). Hence, the annexin V/PI assay was used to evaluate the induction of apoptosis by compounds **8**, **15**, and **16** (20 µM) (**Figure 2**). The results were presented as total apoptosis (early and late apoptotic events) and the effects were expressed as fold increase (ratio between treated samples and untreated samples).

As described above, epoxyboetirane P (**8**) not only presented a highly antiproliferative profile against EPG85-257 gastric cells, but also produced a CS effect for EPG85-257RDB (**Table 1**). After 48 h incubation, compound **8** elicited the activation of caspase-3 in EPG85-257P, EPG85-257RNOV and EPG85-257RDB (**Figure 2A**). Contrary to what would be expected this effect was significantly more pronounced (7-fold) on the parental cell line. With such results, it was expected to observe early and late apoptotic events, after 72 h incubation; however significant results were only obtained for the EPG85-257RNOV cells (**Figure 2A**). Furthermore, epoxyboetirane P (**8**) induced a different response in EPP85-181 pancreatic cells in respect to active caspase-3 (**Figure 2B**). Statistically significant discrimination between resistant pancreatic cell lines and EPP85-181P cells was observed (**Figure 2B**). Compound **8** produced an 8- and 5-fold increase of active caspase-3 in EPP85-181RNOV and EPP85-181RDB cells, respectively. Nonetheless, this compound was not able to induce early or late apoptotic events that would be quantifiable by the annexin V/PI assay, after 72 h incubation.

On our previous studies, methoxyboetirane B (**15**) was highlighted as a promising lead compound for MDR-reversal, since it showed a remarkable ABCB1 modulatory activity potential through a competitive mechanism of action (Matos et al., 2015). Herein, it presented an interesting CS effect on both gastric MDR phenotypes (RR = 0.47 and 0.27, respectively, to EPG85-257RNOV and EPG85-257RDB. These observations were also reflected in the results obtained from the annexin V/PI assays, where a significant stimulation of apoptotic events was recorded in the MDR phenotypes but not in the parental (**Figure 2C**). Compound **15** caused a 3- and 5-fold increase of total apoptosis in EPG85-257RNOV and EPG85-257RDB, respectively. Moreover, the active caspase-3 assays quantified induction of these proteins on about 5.5 to 7-fold, but without significant phenotypic discrimination (**Figure 2C**). In light of these experimental data it might be inferred that the selective antiproliferative activity of methoxyboetirane B (**15**) proceeded through caspase-dependent apoptosis. Regarding the pancreatic cancer cells, compound **15** did not presented CS effect despite the strong antiproliferative effect. Nevertheless, methoxyboetirane B (**15**) showed to be able to provoke differential activation of capase-3 in the MDR phenotypes, but not in the parental (**Figure 2D**). Though, no significant apoptotic events were recorded.

Methoxyboetirane C (**16**) differs from methoxyboetirane B (**15**) on the benzoyl ester substituent, where compound **16** bears a *p*-trifluoromethyl group. This structural variance had impact on reducing the ABCB1 modulatory efficacy of methoxyboetirane C (**16**), when compared with compound **15** (Matos et al., 2015). However, in the present study, structure activity effects were not observed. Both compounds presented comparable antiproliferative profiles against the tested cell lines. Likewise, in the apoptosis induction assays similar effects can be observed (**Figures 2E, F** versus **Figures 2C, D**). Therefore, on gastric EPG85-257RNOV and EPG85-257RDB cells methoxyboetirane C (**16**) modulated a MDR-selective cell death through capase-3-dependent apoptosis (**Figure 2E**). Besides, compound **16** triggered differential activation of capase-3 on pancreatic EPP85-181RNOV and EPP85-181RDB cells, but not in the parental (**Figure 2F**), without significant phosphatidylserine translocation and/or membrane damage.

### Regression Models

In an attempt to identify which molecular descriptors explain the CS effect by the compounds, a computational approach was undertaken by developing regression models, which are powerful tools that allow further insights on the molecular determinants underlying any observed biological activity. They were calculated for a small library of macrocyclic diterpenes, including compounds **1**–**16** along with compounds **17**–**42** (Supporting Information, **Table S1**). For classification purposes, a binary classification identifying the presence (RR ≤ 0.5) or absence (RR > 0.5) of CS was used. Two models were built: the first using a Näive Bayes (NB) classification scheme, a simple probabilistic classifier that examines all samples independently and calculates the individual probability of each particular compound to belong to a distinct cluster (John and Langley, 1995); and a Random Tree (RT) model in which an algorithm constructs trees with K randomly chosen attributes at each node to estimate class probabilities (Breiman, 2001). Herein, both models performed well in predicting which compounds trigger CS in EPG85-257RDB cells, with RT correctly classifying 100% of the compounds against 92.86% with the NB classifier. However, from both the 10-fold cross-validation and internal test (66% training, remaining test set) validations, the NB model provides increased robustness when compared with the RT approach, correctly classifying 85.71% in both validations (MAE, 0.1574 and 0.1106; RMSE, 0.3769 and 0.2685, respectively) against 80.95% (MAE, 0.1905; RMSE, 0.4364) and 85.71% (MAE 0.1429; RMSE, 0.3780) for the RT classifier, respectively (**Table 5**). At the end, we concluded that both models are reliable in predicting which compounds are able to induce CS in the tested P-gp-expressing resistant cancer cell line.

**Table 5:** Data obtained from the herein developed Regression Models.

		TP rate	FP Rate	Precision	Recall	F-Measure	MCC	ROC Area	PRC Area	Class*
**NB**	Train	0.923	0.069	0.857	0.923	0.889	0.973	0.973	0.940	0
		0.931	0.077	0.964	0.931	0.947	0.838	0.973	0.990	1
	10-fold	0.769	0.103	0.769	0.769	0.769	0.666	0.854	0.737	0
		0.897	0.231	0.897	0.897	0.897	0.666	0.854	0.927	1
	Test	0.800	0.111	0.800	0.800	0.800	0.689	0.978	0.967	0
		0.889	0.200	0.889	0.889	0.889	0.689	0.978	0.989	1
**RT**	Train	1.000	0.000	1.000	1.000	1.000	1.000	1.000	1.000	0
		1.000	0.000	1.000	1.000	1.000	1.000	1.000	1.000	1
	10-fold	0.538	0.069	0.778	0.538	0.636	0.529	0.735	0.562	0
		0.931	0.462	0.818	0.931	0.871	0.529	0.735	0.809	1
	Test	0.600	0.000	1.000	0.600	0.750	0.701	0.800	0.743	0
		1.000	0.400	0.818	1.000	0.900	0.701	0.800	0.818	1

*Class 0: RR > 0.5; Class 1: RR ≤ 0.5.

To further improve the ability of the compounds in inducing such a biological response, the identification of the underlying molecular features is of the utmost importance. As in the Naïve Bayes all attributes are assumed to be equally important *a priori* (Lee et al., 2011), from the RT model it is possible to infer which molecular descriptors have the greatest weight in the decision tree. The first and most important descriptor was R4p (R autocorrelation of lag 4/weighted by polarizabilities), followed by MATS8m (Moran autocorrelation of lag 8/weighted by mass) at its first branch and X1A (Average connectivity index of order 1) on the second branch. Together, they are responsible for the classification of 24 compounds with RR ≤ 0.5 (**Figure S1**). For the classification of the remaining compounds, the second branch is further divided by using E2m (2^nd^ component accessibility directional WHIM index/weighted by mass), MDEC-22 (Molecular distance edge between all secondary carbons) and Mor03m (signal 03/weighted by mass) (Todeschini and Consonni, 2009; Devinyak et al., 2014). Herein, it is worth noticing that the E2m descriptor alone is responsible for the classification of 13 compounds (**Figure S1**). All these are geometrical descriptors encode information about the molecular size, shape, geometry, and symmetry. Therefore, it appears that the spatial position of the structural fragments and atom distribution within the molecular scaffold, together with its axial symmetry, are the most important structural determinants ruling CS effects.

Therefore, the aromaticity of the substituent increases the polarizability within the scaffold while promoting CS, in opposition to non-aromatic substituents as in compounds **12**–**14** and **39** (**Table S1**). However, substituents with higher mass ratios, obtained through the quotient between the substituents’ and the molecules molecular weight (as in compounds **3**, **4, 20**, **21**, **37**, and **38**) or with a *meta* substitution pattern most (particularly in mono-substituted compounds **18** and **32**) (**Table S1**) are expected to perturb the axial symmetry of the molecular scaffold and thus impairing its biological activity. Yet, from the data it is also inferred that π-π stacking (**9**, **10**, and **15**) or the presence of additional hydrogen-bond acceptor moieties (as in compounds **8**, **11**, and **16**) are also important for the observed activity.

Overall, the achieved results hints that the substitution pattern and the presence of heteroatoms in an aromatic substituent are the strongest determinants relating to the observed CS effect. Furthermore, as the presence of substituents at positions C-15 and C-17 also impairs CS in a greater extent, the data suggests that future derivatives will have improved CS activities if i) the hydroxyl (or acetyl) group remains unchanged at position C-15 and ii) if only positions C-3 and/or C-5 are substituted but not C-17.

## Conclusion

Targeting more than one anti-MDR mechanism has been considered a realistic strategy for overcoming the complex and multifactorial phenomenon of MDR. In a previous work, some epoxylathyrane derivatives were identified as strong P-glycoprotein efflux modulators. Consequently, aiming at improving their MDR-modifying activity, in this work the CS effect, one of the most hopeful anti-MDR strategies, was addressed. Derivatives **8**, **15**, and **16** were found to be very promising compounds, being many-fold more effective against MDR sublines, mainly in relation to gastric carcinoma cells. In these drug resistant counterparts compounds **15** and **16** significantly induced cell death through apoptosis.

The development of regression models emphasized axial symmetry of the overall molecular scaffold and, more particularly, its substitution pattern as the most determinant features related to the observed CS effect. Furthermore, while the hydroxyl function at position C-15 (or a small acetyl moiety) seems to be determinant, the aromaticity of the substituent together with the presence of heteroatoms was also inferred to be relevant for the observed activity.

All in all, this study reinforces the potential of macrocyclic diterpenes as leads for the development of MDR-modifying agents.

## Data Availability Statement

All datasets generated for this study are included in the article/**Supplementary Material**.

## Author Contributions

All authors have participated and contributed substantially to this manuscript. HL and M-JF designed and supervised the study. MR, AM, and OA carried out the experiments. RF performed the regression models. M-JF, MR, ND, and RF wrote the manuscript. M-JF, MR, and ND revised the manuscript.

## Funding

This study was financially supported by the FCT (Fundação para a Ciência e a Tecnologia) under the project PTDC/MED-QUI/30591/2017 and SAICTPAC/0019/2015.

## Conflict of Interest

The authors declare that the research was conducted in the absence of any commercial or financial relationships that could be construed as a potential conflict of interest.

## Abbreviations

ABC, ATP binding cassette; ABCB1, ATP-binding cassette, sub-family B; CS, collateral sensitivity; EPG85- 257P, parental gastric cancer cells; EPG85- 257RDB, gastric cancer cells selected against daunorubicin; EPG85- 257RNOV, gastric cancer cells selected against mitoxantrone; EPP-181 RDB, pancreatic cancer cells selected against daunorubicin; EPP-181P, parental pancreatic cancer cells; EPP-181RNOV, pancreatic cancer cells selected against mitoxantrone; MDR, multidrug resistance; *MDR1*, multidrug resistance gene 1; RR, relative resistance; NB, Naïve Bayes; RT, random trees.
