# Supplementary material for: Epoxylathyrane Derivatives as MDR-Selective Compounds for Disabling Multidrug Resistance in Cancer
Source: Front Pharmacol. 2020 May 8;11:599. doi: 10.3389/fphar.2020.00599 (PMC7226783; doi:10.3389/fphar.2020.00599)
Supplement: Supplementary file 1 [file DataSheet_1.pdf]

Supplementary Material

Epoxylathyrane derivatives as MDR-selective compounds for disabling multidrug resistance in cancer

Mariana Alves Reis[1,3#], Ana M. Matos[1#], Noélia Duarte[1], Omar Bauomy Ahmed[2], Ricardo Ferreira[1,4], Hermann Lage[2] and Maria-José U Ferreira[1, *]

[1]Research Institute for Medicines (iMed.ULisboa), Faculty of Pharmacy, Universidade de Lisboa, Avenue Prof. Gama Pinto, 1649-003 Lisbon, Portugal

[2] Institute of Pathology, University Hospital Charité, 10117 Berlin, Germany

[3]Current address: Interdisciplinary Centre of Marine and Environmental Research (CIIMAR/CIMAR), University of Porto, Terminal de Cruzeiros do Porto de Leixões, Avenida General Norton de Matos, S/N, 4450-208 Matosinhos, Portugal

[4]Science for Life Laboratory, Department of Cell and Molecular Biology, Uppsala University, 75124 Uppsala, Sweden

[#]First co-authors

*** Correspondence:** Maria-José U. Ferreira

mjuferreira@ff.ulisboa.pt

Table of contents

Materials and Methods……………………………………………………………………S2

Cell Lines, Cell Culture………………………………………………………………….. S2

Cell Proliferation Assay, Annexin V/PI Staining and Active Caspase-3 Assay……………………..S2

Tables and Figures………………………………………………………………………S4

Table S1. Macrocyclic diterpenes included in the dataset………………………………………...S4

Table S2. Characteristics of cancer cell lines and drug-resistant sublines…………………………..S8

Figure S1. Classification tree generated using the Random Tree model…………………………...S10

Materials and Methods

Cell Lines, Cell Culture

The establishment and characterization of the human carcinoma cell lines and drug-resistant sublines used have been described previously (Table S2; Reis et al., 2014). The human cancer cell lines (EPG85-257P gastric; EPP85-181P pancreatic; and HT-29P, colon) and their drug-resistant sublines (EPG85-257RNOV, EPG85-257RDB, EPP85-181RNOV, EPP85-181RDB, HT-29RNOV, HT-29RDB) were grown in Leibovitz L-15 medium (Biowhittaker, Walkersville, MD, USA). All were supplemented with 10% fetal calf serum (FCS) (GIBCO/BRL, GrandIsland, NY, USA), 1 mM L-glutamine, 6.25 mg/L fetuin, 80 IE/L insulin, 2.5 mg/mL, transferrin, 0.5 g/L glucose, 1.1 g/L $NaHCO_3$, 1% minimal essential vitamins, and 20,000 kIE/L trasylol. The cultures were maintained in a humidified atmosphere of 5% CO_2 at 37 ºC. Drug-resistant cell lines were established from parental cell lines by continuous exposure of the cells to stepwise increasing concentrations of antineoplastic agents. For maintenance of drug-resistant phenotypes, the medium of drug-resistant sublines was supplemented with the anticancer agents mitoxantrone and daunorubicin.

Cell Proliferation Assay, Annexin V/PI Staining and Active Caspase-3 Assay

The antiproliferative activity of compounds was evaluated using a proliferation assay based on sulforhodamine B (SRB) staining as previously described (Reis et al., 2014). Briefly, 800 cells per well were seeded in 96-well plates in triplicates. After 24h attachment, the particular agent was added in a dilution series for 5 days incubation (5 % CO_2 at 37 ºC). Cells were fixed by chilled 10% trichloroacetic acid for 1 h at 4 ºC, washed five times with tap water before staining was performed with 0.4 % SRB in 1 % acetic acid for 10 min at room temperature. After washing with 1% acetic acid, drying and resolubilization in 20 mM Tris-HCl (pH10), absorbance was measured at 562 nm against the reference wavelength of 690 nm. Etoposide (≥ 98; Sigma) and cisplatin (Sigma) were used as positive control. Mean IC_{50}-values and standard deviations were calculated from four independent experiments in triplicate for each cell line by using the Prism software (GraphPad Software, Inc.; San Diego, CA, USA). Relative resistance (RR) values were also determined as: $IC_{50(\text{resistant cells})}/IC_{50(\text{parental cells})}$.

For detection of cytotoxic drug-induced apoptosis, a FITC Annexin V apoptosis detection kit (BD Pharmingen, BD Biosciences) was used. Detection of intracellular presence of active caspase-3 was

also performed using FITC active Caspase-3 Apoptosis Kit (BD Pharmingen', BD Biosciences). Both assays followed the same experimental design. Briefly, 6×104 cell/ml of parental cell lines (EPG85-256P and EPP85-181P) and 1 × 105cell/ml of resistant cell lines (EPG85-256RNOV, EPG85-256RDB, EPP85-181RNOV and EPP85-181RDB) were seeded in six-well plates in complete medium and allowed to attach for 24 h. On the next day, the medium was discarded and new medium, with 30 µM of the tested compounds, was added to the gastric and pancreatic cancer cells. Final concentration of DMSO in experiment was 0.3% (v/v). Camptothecin (Cayman Chemicals, USA) was used as positive control (1 µM). Cells were further incubated for 72 h, in 5% CO2 at 37 ºC. The concentration of compounds and positive control and incubation time were optimized in order to assure a good sampling for flow cytometry measurement of the apoptotic process (data not shown). After this incubation period, cells were trypsinized, washed in PBS and stained according to each kit manufacturer's instructions. Stained cells were analyzed using BD Accuri C6 flow cytometer (BD Pharmingen', BD Biosciences) and data were processed with BD Accuri C6 software. Each sample was assessed using a collection of 10 000 events. The mean values and standard deviations were calculated from three independent experiments.

Statistical evaluation of the apoptosis assays data was performed with the two-tailed unpaired Student's *t* test using GraphPadPrism5 software. Probability value $p < 0.05$ was considered statistically significant.

Tables and Figures

Table S1. Macrocyclic diterpenes included in the dataset.

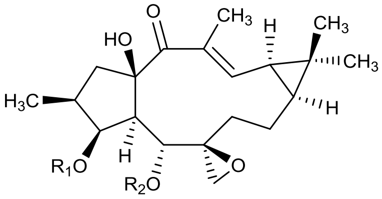

Compounds **1-12**

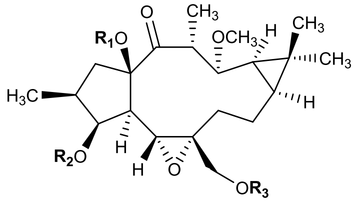

Compounds **13-16**

Compound	R1	R2	R3
Epoxylathyrol (**1**)	H	H	---
Epoxyboetirane E (**2**)	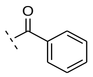	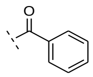	---
Epoxyboetirane K (**3**)	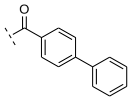	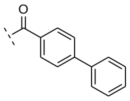	---
Epoxyboetirane L (**4**)	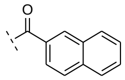	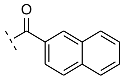	---
Epoxyboetirane M (**5**)	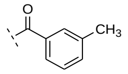	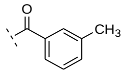	---
Epoxyboetirane N (**6**)	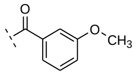	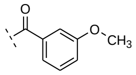	---
Epoxyboetirane O (**7**)	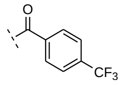	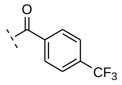	---
Epoxyboetirane P (**8**)	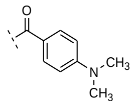	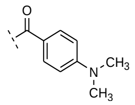	---
Epoxyboetirane Q (**9**)	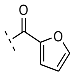	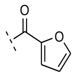	---
Epoxyboetirane R (**10**)	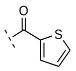	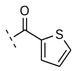	---
Epoxycarbamoylboetirane B (**11**)	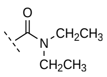	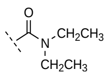	---
Epoxycarbamoylboetirane C (**12**)	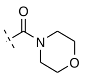	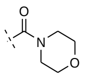	---
Methoxyboetirol (**13**)	H	H	H

Methoxyboetirane A (**14**)	H	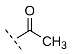	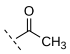
Methoxyboetirane B (**15**)	H	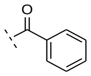	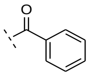
Methoxyboetirane C (**16**)	H	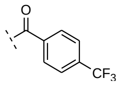	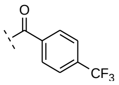

Table S1. (continued)

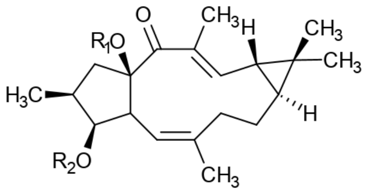

Compounds **17-41**

**Compound**	**R1**	**R2**
Jolkinodiol (**17**)	H	H
Jolkinolate A (**18**)	H	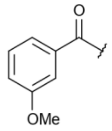
Jolkinolate B (**19**)	H	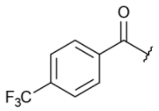
Jolkinolate C (**20**)	H	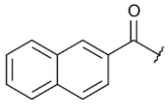
Jolkinolate D (**21**)	H	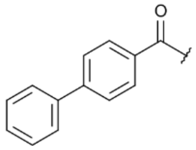
Jolkinol D (**22**)	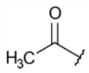	H
Jolkinoate A (**23**)	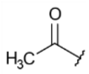	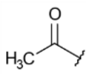
Jolkinoate C (**24**)	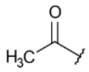	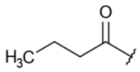

Jolkinoate D (**25**)

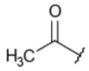

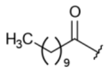

Jolkinoate E (**26**)

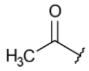

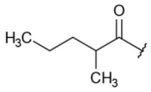

Jolkinoate G (**27**)

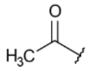

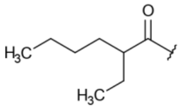

Jolkinoate I (**28**)

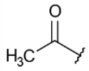

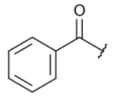

Jolkinoate K (**29**)

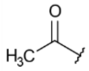

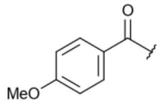

Jolkinoate L (**30**)

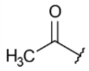

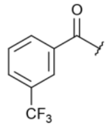

Jolkinoate M (**31**)

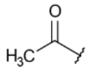

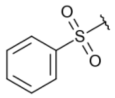

Jolkinoate N (**32**)

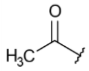

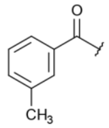

Jolkinoate O (**33**)

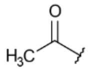

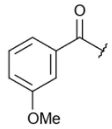

Jolkinoate P (**34**)

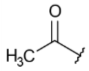

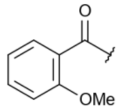

Jolkinoate Q (**35**)

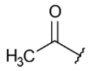

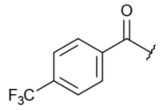

Jolkinoate R (**36**)

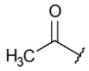

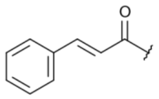

Jolkinoate S (**37**)

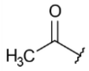

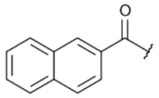

Jolkinoate T (**38**)	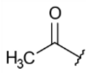	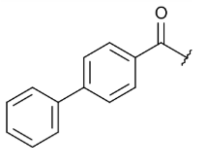
Jolkinoate U (**39**)	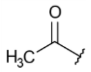	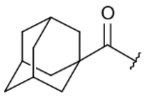
Jolkinocarbonate A (**40**)	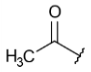	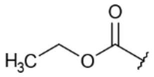
Jolkinocarbonate B (**41**)	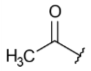	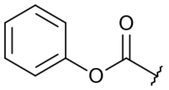
Esulatin M (**42**)	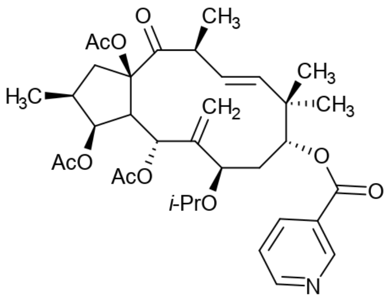	

Table S2. Characteristics of cancer cell lines and drug-resistant sublines

* To ensure the MDR phenotype the growth medium of each cell line is supplemented with a determined concentration of selection agent.

Origin	Gastric carcinoma				Pancreatic carcinoma				Colon carcinoma			
Cell line	EPG85-257P	EPG85-257RN	EPG85-257RD		EPP85-181P	EPP85-181RN	EPP85-181RD		HT-29P	HT-29RN	HT-29RD	
Selection agent*	-[1]	Mitoxantrone (0.2 µg/ml)[1]	Daunorubicin (2.5 µg/ml)[1]		-[2]	Mitoxantrone (0.02 µg/ml)[2]	Daunorubicin (2.5 µg/ml)[2]		-[3]	Mitoxantrone (0.2 µg/ml)[3]	Daunorubicin (0.25 µg/ml)[3]	
	mRNA expressing level			Ref	mRNA expressing level			Ref	mRNA expressing level			Ref
MRP1	+	+	+	4	+	+	+	4	+	+	+	4
MRP2	+	+↑	+↑	4	+	+↑	+↑	4	+	+↑	+↑	4
MRP3	-	-	-	4	+↓	+↑	+↑	4	+	+↑	+	4
MRP4	+	+↑	+↑	4	+	+↑	+↑	4	+	+↑	+↑	4
MRP5	+	+↑	+↑	4	+	+↑	+↑	4	+	+↑	+↑	4
MRP6	-	-	-	4	+	-	-	4	+	-	+↑	4
MRP7	+	+	+	4	+	+	+	4	+	+	+	4
MRP8	+↓	-	-	4	-	-	-	4	-	-	-	4
MRP9	-	-	-	4	-	-	-	4	-	-	-	4
MDR1	-	-	+↑	4	-	-	+↑	2,4	-	-	-	4
BCRP	+↓	+↑	+↓	4,5	-	-	-	2,4	+↓	+↓	+↓	4
GPC3	+↓	+↑		6,7								
TAP	+	+↑	+	8								
TOPO II	+	+↓		9	+	+↓	+↓	10				

MRP = multidrug resistance protein; MDR1 = P-gp; BCRP = breast cancer resistance protein; GPC3 = gtypican-3; TAP = transporter associated protein; TOPO = DNA topoisomerase; + = mRNA expressed; +↓ = low levels of mRNA expressed; +↑ = high levels of mRNA expressed; ; - = no expression.

References of the Table S2

1. Dietel, M., Arps, H., Lage, H. & Niendorf, A. Membrane Vesicle Formation Due to Acquired Mitoxantrone Resistance in Human Gastric Carcinoma Cell Line EPG85-257 Membrane Vesicle Formation Due to Acquired Mitoxantrone Resistance in Human Gastric Carcinoma Cell Line EPG85-2571. 6100–6106 (1990).
2. Lage, H., Jordan, a, Scholz, R. & Dietel, M. Thermosensitivity of multidrug-resistant human gastric and pancreatic carcinoma cells. *Int. J. Hyperthermia* **16**, 291–303 (2000).
3. Sinha, P. *et al.* Search for novel proteins involved in the development of chemoresistance in colorectal cancer and fibrosarcoma cells in vitro using two- dimensional electrophoresis , mass spectrometry and microsequencing Proteomics and 2-DE. (1999).
4. Lage, H., Duarte, N., Coburger, C., Hilgeroth, A. & Ferreira, M. J. U. Antitumor activity of terpenoids against classical and atypical multidrug resistant cancer cells. *Phytomedicine* **17**, 441–448 (2010).
5. Ross, D. D. *et al.* Atypical Multidrug Resistance: Breast Cancer Resistance Protein Messenger RNA Expression in Mitoxantrone-Selected Cell Lines. *JNCI J. Natl. Cancer Inst.* **91**, 429–433 (1999).
6. Lage, H. & Dietel, M. Cloning and characterization of human cDNAs encoding a protein with high homology to rat intestinal development protein OCI-5. *Gene* **188**, 151–6 (1997).
7. Wichert, A., Stege, A., Midorikawa, Y., Holm, P. S. & Lage, H. Glypican-3 is involved in cellular protection against mitoxantrone in gastric carcinoma cells. *Oncogene* **23**, 945–55 (2004).
8. Lage, H. *et al.* Enhanced expression of human ABC-transporter tap is associated with cellular resistance to mitoxantrone. *FEBS Lett.* **503**, 179–84 (2001).
9. Kellner, U. *et al.* Decreased drug accumulation in a mitoxantrone-resistant gastric carcinoma cell line in the absence of P-glycoprotein. *Int. J. Cancer* **71**, 817–24 (1997).
10. Lage, H. & Dietel, M. Multiple mechanisms confer different drug-resistant phenotypes in pancreatic carcinoma cells. *J. Cancer Res. Clin. Oncol.* **128**, 349–57 (2002).

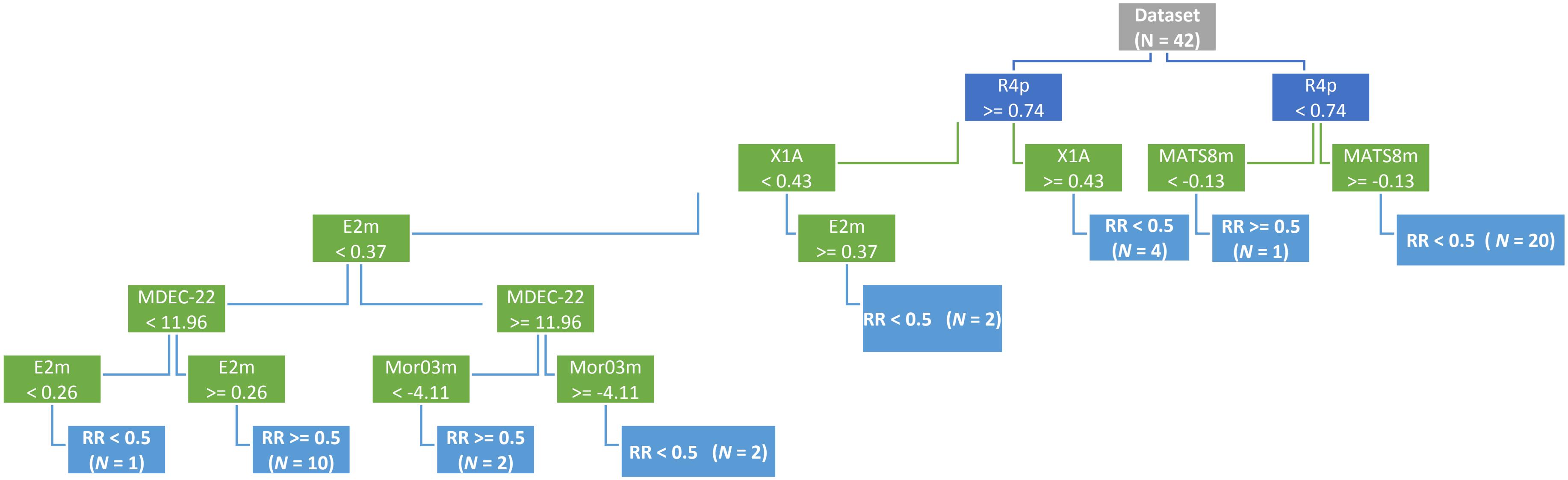

Figure S1. Classification tree generated using the Random Tree model.

References (for compounds **17-42**)

Reis, M.A., Paterna, A., Ferreira, R.J., Lage, H., Ferreira, M.J.U. (2014). Macrocyclic diterpenes resensitizing multidrug resistant phenotypes. *Bioorg. Med. Chem.* 22, 3696–3702.

Reis M.A., Ahmed, O.B., Spengler, G., Molnár, J., Lage, H., Ferreira, M.J. U. (2016). Jatrophane diterpenes and cancer multidrug resistance - ABCB1 efflux modulation and selective cell death induction. *Phytomedicine* 23, 968-978.

Reis M.A., Ahmed, O.B., Spengler, G., Molnár, J., Lage, H., Ferreira, M.J. (2017). Exploring Jolkinol D derivatives to overcome multidrug resistance in cancer. *J. Nat. Prod.* 80, 1411-1420.
